# First reported case of thymoma‐associated multiorgan autoimmunity induced by COVID‐19

**DOI:** 10.1111/1346-8138.17519

**Published:** 2024-10-26

**Authors:** Rie Hyobu, Miho Mori, Tatsuo Maeda, Kazuki Fujimori, Yukako Shimai, Makiko Naito, Masayuki Masuda, Tatsuo Ohira, Norihiko Ikeda, Yukari Okubo, Kazutoshi Harada

**Affiliations:** ^1^ Department of Dermatology Tokyo Medical University Tokyo Japan; ^2^ Department of Neurology Tokyo Medical University Tokyo Japan; ^3^ Department of Surgery Tokyo Medical University Tokyo Japan

**Keywords:** alopecia, COVID‐19, graft‐versus‐host disease, myasthenia gravis, thymoma

## Abstract

Thymoma‐associated multiorgan autoimmunity (TAMA) presents with skin symptoms similar to those of graft‐versus‐host disease (GVHD), liver dysfunction, and enteritis, in the absence of a history of hematopoietic stem cell or bone marrow transplantation. TAMA is a type of paraneoplastic syndrome associated with thymoma. Its etiology is unclear but is thought to be a result of breakdown of immune tolerance. Histopathologically, TAMA is characterized by epidermal acanthosis with parakeratosis, individual cell keratinization, liquefaction degeneration, and intraepidermal infiltration of CD8‐positive lymphocytes. A 64‐year‐old female patient with a history of myasthenia gravis and thymoma treated with prednisolone (10 mg/day) and cyclosporine (150 mg/day) experienced erythema on her trunk after coronavirus disease 2019 (COVID‐19) onset. A psoriatic drug eruption was suspected and the possible causative drug was discontinued, but the skin rash failed to improve. A skin biopsy demonstrated GVHD‐like histopathological findings. Diarrhea, abdominal pain, and duodenal perforation occurred concurrently, leading to the diagnosis of TAMA. Thereafter, the patient continued prednisolone and cyclosporine in the same doses as the TAMA treatment and added topical steroids. During the disease course, candida fungemia and cytomegalovirus infection developed, resulting in the patient's death. The TAMA was considered to have been caused by the release of inflammatory cytokines, autoreactive T cell activation, and regulatory T cell dysfunction induced by COVID‐19.

## 1 INTRODUCTION

Thymoma‐associated multiorgan autoimmunity (TAMA) is a paraneoplastic syndrome characterized by skin symptoms, liver dysfunction, and gastrointestinal disorders similar to those of graft‐versus‐host disease (GVHD) in the absence of a history of hematopoietic stem cell or bone marrow transplantation.^1^, ^2^ Here, we report a case of TAMA that was thought to have been triggered by COVID‐19.

## 2 CASE REPORT

A 64‐year‐old female Japanese patient presented with erythema on her trunk. Myasthenia gravis (MG) and invasive thymoma (Type B2 according to the World Health Organization (WHO) classification, Masaoka Classification Stage II) had been diagnosed at the age of 48 years. Despite extensive therapy, the tumor recurred multiple times. At the first visit, the patient received prednisolone (10 mg/day), cyclosporine (150 mg/day) as well as pyridostigmine bromide, cilnidipine, and azilsartan.

**FIGURE 1:**
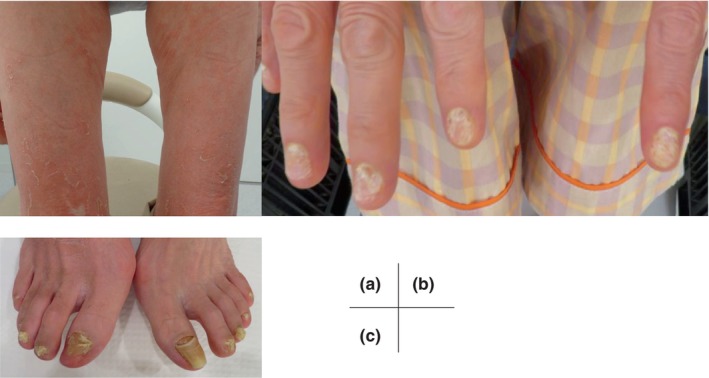
Clinical picture at the initial diagnosis. (a) Erythema of the thigh with desquamation. (b) Nails of both hands showing coarse, degraded nail plates. (c) Toenails with coarse, degraded nail plates.

Her medical history revealed that 6 days after COVID‐19 onset, erythematous plaques with slightly itchy scales up to 10 mm in size appeared on the trunk. Viral exanthema was diagnosed at the initial consultation and was treated with topical steroids without effect. The erythema spread to the extremities (Figure 1a). The coarse and degraded state of the nails of all the fingers and toes led to the suspicion of a psoriatic drug eruption (Figure 1b,c). Histological findings of the erythema on the thighs were consistent with a psoriatic drug eruption.

**FIGURE 2:**
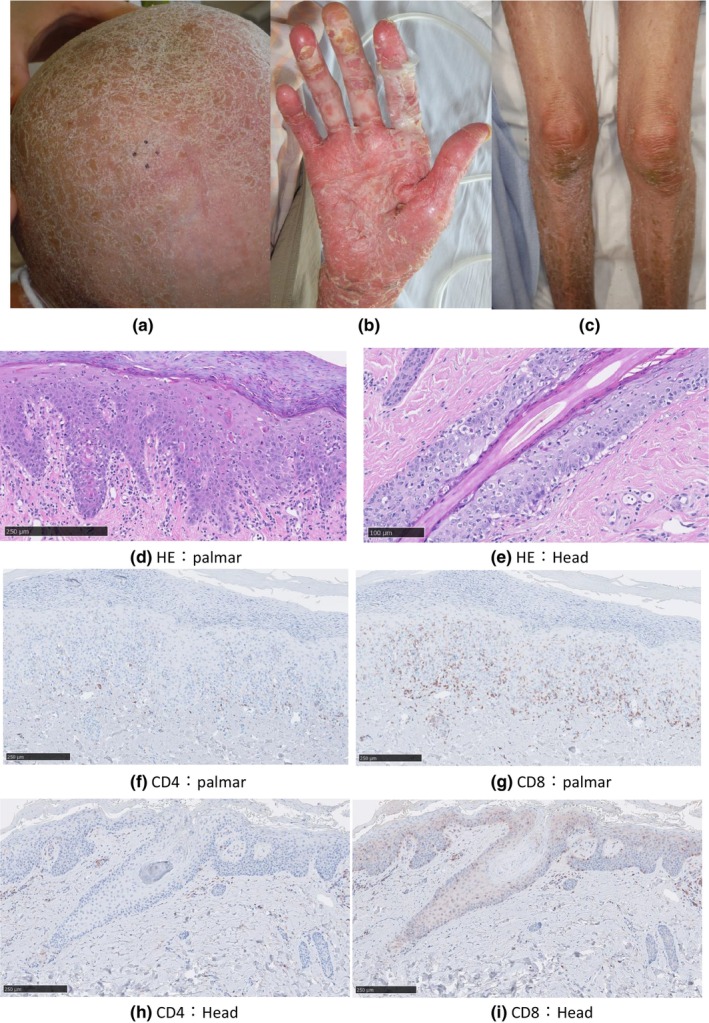
Clinical picture at the time of the second biopsy. (a) Head with frontal‐type alopecia and conspicuous desquamation. (b) Skin biopsy of the palm skin showing marked keratinization and desquamation. (c) Erythema of the lower extremities showing healing and desquamation. (d) Palm skin showing hyperkeratosis with parakeratosis and epidermal intercellular edema. Vacuolar degeneration of the epidermal‐dermal border and lymphocytic infiltration into the epidermis were observed. Keratinization of numerous cells was observed in the epidermis. Lymphocytic infiltrate was found in the shallow dermis (hematoxylin–eosin [H‐E] original magnification, ×200). (e) Scalp skin demonstrated epidermal and dermal findings similar to those of the palms. Marked lymphocytic infiltration in the follicular epithelium and keratinization of numerous cells were observed (H‐E original magnification, ×200). (f) CD4 staining of the palm skin revealed sparse infiltration of CD4‐positive T cells in the epidermis. (g) CD8 staining of the palm skin revealed marked intra‐epidermal infiltration of CD8‐positive T cells. (h) CD4 staining of the scalp skin revealed a small number of CD4‐positive T cells in the epidermis. (i) CD8 staining of the scalp skin revealed numerous CD8‐positive T cells infiltrating the follicular epithelium. Markedly more CD8‐positive than CD4‐positive cells had infiltrated the epidermis of the palms and scalp (original magnification, ×200).

Cilnidipine and azilsartan, which were thought to be causative drugs,^3^ were discontinued. The patient received topical steroids, but 2 months later, in addition to deterioration of the rash on the trunk, keratinization of the soles and palms became evident. Abdominal pain and diarrhea also occurred at around the same time. In addition, the patient had been experiencing scalp hair loss from 1 year before the initial visit and had subsequently developed total alopecia. She had no history of bone marrow transplantation, but based on her clinical symptoms, GVHD was suspected. Biopsies of the scalp and palm skin were, therefore, performed again (Figure 2a–c).

A second biopsy of the palm skin demonstrated hyperkeratosis with parakeratosis, epidermal intercellular edema, lymphocytic infiltration in the epidermis, and individual cell keratinization. Vacuolar degeneration at the epidermal–dermal border and a lymphocyte‐dominated inflammatory cell infiltrate in the shallow dermis were also observed (Figure 2d). The scalp biopsy findings were almost identical to a previous examination (Figure 2e). Immunohistochemistry revealed a greater number of CD8‐positive cells than CD4‐positive cells in the epidermal infiltrates of the bilateral palms as well as the scalp (Figure 2f–i).

Based on the history of invasive thymoma without a history of hematopoietic stem cell or bone marrow transplantation, gastrointestinal disorders, and histopathological findings mimicking GVHD, TAMA was diagnosed. Prednisolone (10 mg/day) and cyclosporine (150 mg/day), which had been administered before TAMA onset, were continued in the same doses. In the clinical course, abdominal computed tomography (CT) demonstrated free air and duodenal perforation. Endoscopic and conservative treatment were administered in view of the patient's deteriorating general condition. The patient also had a cytomegalovirus (CMV) infection and candida fungemia. She died 3 months later from a disseminated intravascular coagulation caused by the gastrointestinal perforation.

## 3 DISCUSSION

Thymoma‐associated multiorgan autoimmunity was recently categorized as a thymoma‐associated autoimmune disease.^4^ In their analysis of 29 patients with TAMA, Shiba et al.^5^ reported skin symptoms in 72.4% (*n* = 21), enteritis in 48.3% (*n* = 14), and liver dysfunction in 34.5% (*n* = 10) of patients.

A search of PubMed and the Japanese medical journal, *Ichushi*, retrieved 57 previous reports of TAMA with skin symptoms (Table 1). Patients in whom three organs were involved (*n* =10; 18%) all eventually died from complications of the disease. Patients in whom the skin symptoms preceded the detection of a thymoma (*n* = 2), those in whom TAMA was discovered simultaneously with a thymoma (*n* = 4), and those who received chemotherapy for recurrences several years after onset (*n* = 2) reportedly all survived. The fundamental treatment for thymoma was important, but many of the patients (*n* = 32; 56%) had poorly controlled disease due to their poor, general condition or recurrences, and treatment was not always possible. Moreover, 43 patients (75%) had a history of the use of an immunosuppressant agent, such as prednisolone or cyclosporine, before TAMA onset, which probably predisposed them to opportunistic infections. Notably, seven patients (7/37, 19%) who died had a CMV infection. The possibility of a thymoma recurrence could not be ruled out in our patient because a CT‐guided biopsy was not performed to confirm the diagnosis. The skin manifestations of TAMA vary and scaling erythema, keratotic erythema, and papules were reported. The rash may change over time, sometimes presenting with erythroderma or a marked keratosis of the palms and soles. The differential diagnosis includes psoriasis, drug eruption, drug‐induced hypersensitivity syndrome, viral toxicosis, pityriasis rosea Gibert, and pityriasis lichenoides, all of which were ruled out in the present case on the basis of histopathological and blood test findings and a distinctive medical history. Alopecia occurred in seven patients with TAMA. Although the histopathological examination of our patient's scalp demonstrated GVHD‐like findings, the skin biopsy was performed after the onset of TAMA and there are no reports of alopecia preceding GVHD. Hence alopecia may be a symptom of an autoimmune disease related to the thymoma.^6^

**TABLE 1:** Reports of TAMA with skin symptoms.

Sex (M:F) *n* = 57	19:38
Tumor stage	
Stage I	1
Stage II	6
Stage III	4
Stage IV	21
Tumor pathology	
A	1
AB	3
B1	11
B2	13
B3	4
Age at TAMA onset (years)	26 ~ 77 (average 53.5)
Target organ	
Skin	28
Skin+liver	13
Skin+intestine	6
Skin + liver + intestine	10
Period from thymoma diagnosis to TAMA onset (*n* = 51)	
2 months–23 years (average 7.2 years)	44
Skin symptoms preceding	2
Concurrent with diagnosis	5
Recurrence or poor control of thymoma status at TAMA onset	32
Complications (duplicate)	
MG	39
PRCA	11
GS	9
PM	4
SLE	2
Sjögren syndrome	2
RA	2
Vitiligo vulgaris, thyroiditis, hypothyroidism	1 example each
Oral lichen planus, bone marrow aplasia, T cell lymphoma	1 example each
Clinical features	
Erythroderma	18
Palmoplantar papules and keratoplasty	7
Mucous membrane	10
Alopecia (before:after) *n* = 7	2:5
Prognosis (alive:dead) *n* = 52	15:37
Cytomegalovirus infection among death cases (*n* = 37)	7

Abbreviations: GS, good syndrome; MG, myasthenia gravis; PM, polymyositis; PRCA, pure red cell aplasia; RA, rheumatoid arthritis; SLE, systemic lupus erythematosus; TAMA, thymoma‐associated multiorgan autoimmunity.

The etiology of TAMA is thought to result from a breakdown of immune tolerance. Autoimmune regulators (AIRE) expressed on thymic medullary epithelial cells have a role in promoting the expression of tissue‐specific autoantigens and in eliminating autoreactive T cells. In TAMA, AIRE are completely absent in the thymoma, rendering negative selection unresponsive, and the number of regulatory T cells (Tregs) in the thymoma is reduced.^7^ Consistent with this, skin lesions in TAMA reportedly display the same tissue response and marked reduction in infiltrating Tregs as seen in GVHD.^4^

Similarly, the number of Tregs was found to be very low in COVID‐19 patients, potentially weakening the effect of inflammatory suppression.^8^ Several reports revealed an increase in the risk of autoimmune disease development, including rheumatoid arthritis, ankylosing spondylitis,^9^ type 1 diabetes mellitus, inflammatory bowel disease, and psoriasis,^10^ following COVID‐19 onset. Interestingly, some studies have reported that the homology between some human autoproteins and components of SARS‐CoV‐2 may promote the development of autoimmune diseases, and that tissue damage and the release of autoantigens resulting from a SARS‐CoV‐2 infection may trigger a self‐aggressive response.^10^

Moreover, SARS‐Cov‐2 infection reportedly triggers the release of various proinflammatory cytokines, such as interleukin (IL) 6, IL‐1, tumor necrosis factor‐α, and interferon‐γ,^8^ which are involved in the pathogenesis of GVHD.^11^ Indeed, the reactivation of chronic GVHD following COVID‐19 onset has been reported in patients following a hematopoietic stem cell transplantation, suggesting that a viral infection may promote and amplify the abnormal processes in pre‐existing GVHD.^12^ There is also a study reporting histopathological findings of acute GVHD‐like diarrhea after COVID‐19 in patients without a history of thymoma.^13^ This has led to the suggestion that a cytokine storm and immune overload may have caused the GVHD‐like gastrointestinal damage in the patients.^13^

The present case is the first to report TAMA occurrence following COVID‐19 onset. The present patient had raised the suspicion of an association between the latent immune abnormality and COVID‐19, which may have triggered the release of inflammatory cytokines, autoreactive T cell activation, and Treg dysfunction, resulting in TAMA. There are reports of GVHD as well as TAMA^14^ resulting from the activation of a cytotoxic immune response following a herpes zoster infection. Thus, patients with a history of thymoma who contract a viral infection, such as COVID‐19, may be at increased risk of developing TAMA. Furthermore, a link between the pathogenesis of GVHD and herpes viruses has recently been noted,^15^ making it important to monitor patients with TAMA for a potential CMV infection.

The present case report included only a small number TAMA cases which developed after, or were exacerbated by, a viral infection. Thus, further studies are needed to verify the findings.

## CONFLICT OF INTEREST STATEMENT

None declared.
